# Micro-to-nano scale filling behavior of PMMA during imprinting

**DOI:** 10.1038/s41598-017-08409-9

**Published:** 2017-08-11

**Authors:** Jingmin Li, Ziyang Liu, Chao Liang, Xia Li, Jinguang Fan, Hao Zhang, Chong Liu

**Affiliations:** 1 Key Laboratory for Micro/Nano Technology and System of Liaoning Province, Dalian University of Technology, Dalian, China; 2 Key Laboratory for Precision and Non-traditional Machining Technology of Ministry of Education, Dalian University of Technology, Dalian, China

## Abstract

The filling behavior of polymers in narrow gaps or small pores is important for the dynamics of polymeric micro/nanostructure fabrication. Here, the filling behavior, the mechanical properties, and the stress versus strain relationship of 996 kD poly (methyl methacrylate) (PMMA) at a scale from micron to molecular confinement are measured. It has been found that the solid polymer exhibits elastic-plastic dominant deformation behavior at micron scale. As the scale reduces to submicron, the resistance to deformation of the polymeric solid has a pronounced reduction. A softening effect and the visco-dominant behavior which is always exhibited by melt flow is observed. In confinement conditions, an anomalous hardening effect is found. The modulus and the hardness of 996 kD PMMA have been found to increase dramatically. The stress-strain curve also exhibits an obvious hardening phenomenon which is contrary to the conventional shear thinning and deformation acceleration results. The results of this paper show that the PMMA can exhibit a change of “solid-fluid-solid” in mechanical character at micron to molecular confinement scale.

## Introduction

Polymers have become common in the manufacturing of semiconductor devices, organic electronics, optics, and biomedical microdevices due to their ease and economy in processing and mass producing^1, 2^. The most well-known methods to fabricate polymeric micro/nano devices are hot embossing and nanoimprint lithography. They exploit solid polymer deformation or melt polymer flow at micro/nano scale to form patterns. During fabrication, a rigid, patterned die squeezes a supported polymer film. Filling of the die cavities or gratings requires longitudinal and lateral flow of polymer. The change of polymer mechanical properties at different scales has significant effect on the processing conditions and ultimate replication fidelity^3–5^.

As the fabrication temperature is above or near polymeric melting temperature (Tf), the polymer exhibits melt or near-melt mechanical properties which is called “viscous-dominant”^6, 7^, The flow dynamics of viscous-dominant polymer into millimeter to nanometer scale cavities are mainly determined by capillary force, shear-thinning, as well as the buckling^8–10^. Its filling behavior usually exhibits dual-peak viscous flow, which means the polymer flows into the cavity and form two peaks along the cavity sidewalls^11–13^. In contrast, as the imprint temperature is slightly above polymer glass transition temperature (Tg) but far below melting temperature, the polymer still exhibits solid-state mechanical properties which is called “elastic-dominant”^14, 15^. At this state, the pressing may induce localized stress concentration and hardening which can reduce the motion of solid molecular chains. The surface force cannot dominate the deformation of a polymeric solid. As the ratio of cavity depth to cavity width is 1.0, the solid polymer usually exhibits single-peak elastic-plastic deformation during the filling of a millimeter or micron scale cavity^8–13, 16^, which indicates the filling peak forms in the center of the cavity and cannot generate near the cavity side wall surfaces. As the cavity size reduces to nanometer scale, conventional studies still support the issue that elastic-plastic character domains the deformation of solid-state polymer. The filling modes is still “single-peak”. In some cases, dual-peak is observed in a cavity with an aspect ratio far more than 1.0 ^17, 18^. But the form of this type of dual-peak is attributed to the extensional stretching which means there are not enough deformation force to support the form of a peak far from the cavity sidewall. An anomalous fluid-like behavior is found at the molecular confinement scale for polystyrene (PS)^19^. An illustration for those phenomenon is that the surface force and viscous force will dominate the chain motion of polymeric solid at nanometer and molecular confinement scale. Some studies also have found that localized stress concentration can cause rapid shear thinning to enhance the viscous flow. Furthermore, Tg, modulus, as well as forming stress will also have an obvious decrease at this scale.

In this paper, we investigate the mechanical characters and filling modes of solid poly (methyl methacrylate) (PMMA) at the scale from microns to molecular confinement. The filling experiments at the scale from 20 nm to 2 μm are performed to show the influence of size scale on the deformation modes of solid PMMA. The moduli, hardnesses and stress-strain curves at different scales are established to exhibit the effects of scale reduction on PMMA mechanics. An anomalous phenomenon of “solid-fluid-solid” mechanical deformation is observed.

## Experimental Section

### Sample preparation

In this paper, we used two kinds of PMMA materials with molecular weights of 996 kD and 100 kD respectively for experiments. PMMA with average molecular weight (MW) 996 kD and 100 kD was purchased from Sigma-Aldrich, with Tg = 105 °C and density of 1.2 g/cm3. The chain length (h) can be given by^20, 21^

1 $$h=2l\sqrt{{M}_{W}/{M}_{W}^{S}}$$

where *l = 0.153 nm* was bond length, ${M}_{W}^{S}$ was molecular weight and ${M}_{W}^{S}$ *= 100.12D* is the molecular weight of the repeating chain unit. It can be derived that for 996 kD PMMA, h = 30.5 nm, and for 100 kD PMMA h = 9.7 nm. For 996 kD PMMA, as the deformation of polymeric solid was confined within a space less than 30.5 nm, it was a molecular confinement scale deformation. For 100 kD PMMA, as the molecular confinement scale was 9.7 nm. The experimental samples were prepared by spin coating the PMMA solutions (dissolved in chlorobenzene) onto a silicon wafer. For nano-indentation tests, the casting speeds ranged from 2000 to 6000 rpm, the dilution of solution ranged from 0.1% to 1%. The obtained 996 kD PMMA films had thicknesses of 23 nm, 36 nm, 71 nm, 130 nm, 220 nm, 450 nm and 680 nm respectively. The 100 kD PMMA films had thicknesses of 20 nm, 32 nm, 53 nm, 70 nm, 130 nm and 220 nm respectively. For embossing experiments, the casting speed was 1000 rpm-1200 rpm, the film thicknesses were all 6 µm.

### Embossing die

**Figure 1:**
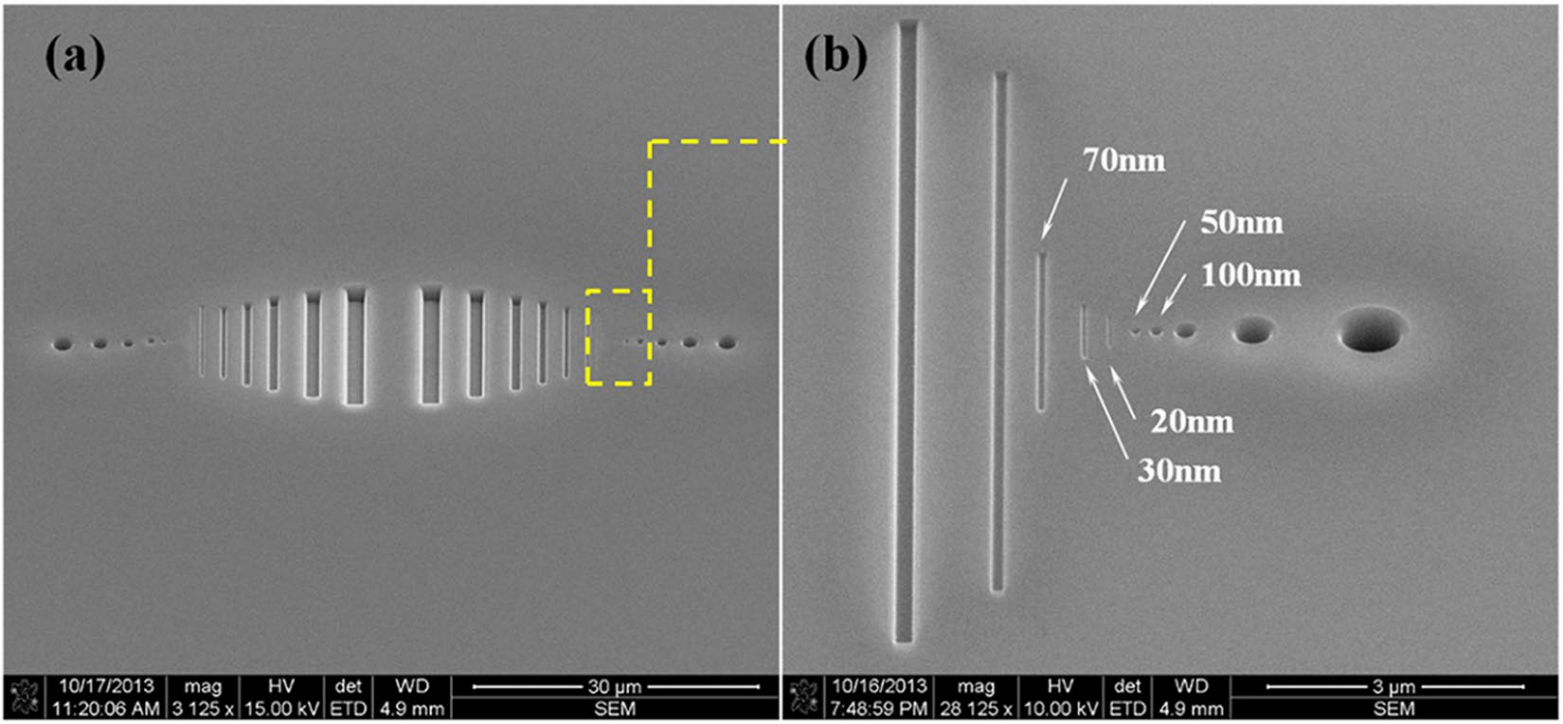
The embossing die. (**a**) The gratings and pores on the die. (**b**) The nanometer gratings and pores on the die.

A glass die was used for filling experiments. The deposition of 2.3 µm TiN layer was performed in Changzhou Institute of Dalian University of Technology. Ion beam was used to etch the gratings and pores on the TiN layer. The etching was done in Tianjin Micronanoelectronic Manufacturing Technology Co., Ltd. Figure 1 shows the features on the die. The features are composed of 20 gratings and 14 pores. All the cavities have an aspect ratio of 1. The widths of the gratings range from include 2.0 µm, 1.6 µm, 1.2 µm, 800 nm, 400 nm, 200 nm, 120 nm, 70 nm, 30 nm and 20 nm. The diameters of the pores include 2.0 µm, 1.6 µm, 1.2 µm, 700 nm, 400 nm, 200 nm, 100 nm and 50 nm. The spaces between the cavities are shown in Fig. 1, which vary from 1.09 µm to 2.93 µm.

The filling of 996 kD PMMA into the cavities of 20 nm and 30 nm were molecular confinement scale deformation due to the size of 20 nm and 30 nm were less than the chain length (h) of 996 kD PMMA. The sizes of 50 nm pore and the 70 nm grating were slightly above h of 996 kD PMMA. Here, we call the filling behavior of PMMA into these two cavities as “near-confinement scale deformation”.

### Micro/nano features replication procedure

The embossing machine was developed by our group. It can provide an imprint pressure of 100 N to 10000 N with an accuracy of 1 N. It uses thermoelectric cooler to heat and cool the samples and the die. The embossing temperature can be ranged from 25 °C to 150 °C. The value of pressure and temperature were recorded and processing by PMAC board and shown in computer through a profile. This research used this machine to replicate the features of the die onto the PMMA samples. The replication processes were as follows. First, the die and the sample are heated to 90 °C and remain for 2–3 min. Second, the die and the sample were heated from 90 °C to 110 °C with a heating rate of 0.2 °C/s. Embossing pressure is applied during the heating process with a rate of 0.02 MPa/s. As the pressure increasesd to 2000 N and the temperature increased to 110 °C, remaining for 2 min.Third, reducing the temperature to 40 °C and decreasing the pressure to 500 N. Re-heating the PMMA sample to about 70 °C, subsequently, releasing the die from the PMMA sample and recording the filling peak mode of PMMA.

### Nano-indentation tests

MTS Nano Identer XP had been used to perform the nanoindentation on the PMMA films with different thicknesses. Two types of indenters, including berkovich tip and sphere tip, were used for indentation. As the stress and strain generated by using a sphere tip can be derived from experimental parameters, such as applied pressure, tip radius, whole indenter radius and pressing depth, we used sphere tip to obtain the stress-strain relationship of a PMMA film in this study. The berkovich tip was used to obtain the hardness and modulus. The operation platform of the indentation machine had been modified to heat the PMMA substrate to a certain temperature. In this experiments, the PMMA substrate had been heated to 110 °C which is the embossing temperature used in normal replication of micro/nano structures.

## Results and Discussion

### Filling Mode from Micron to Molecular Confinement Scale

**Figure 2:**
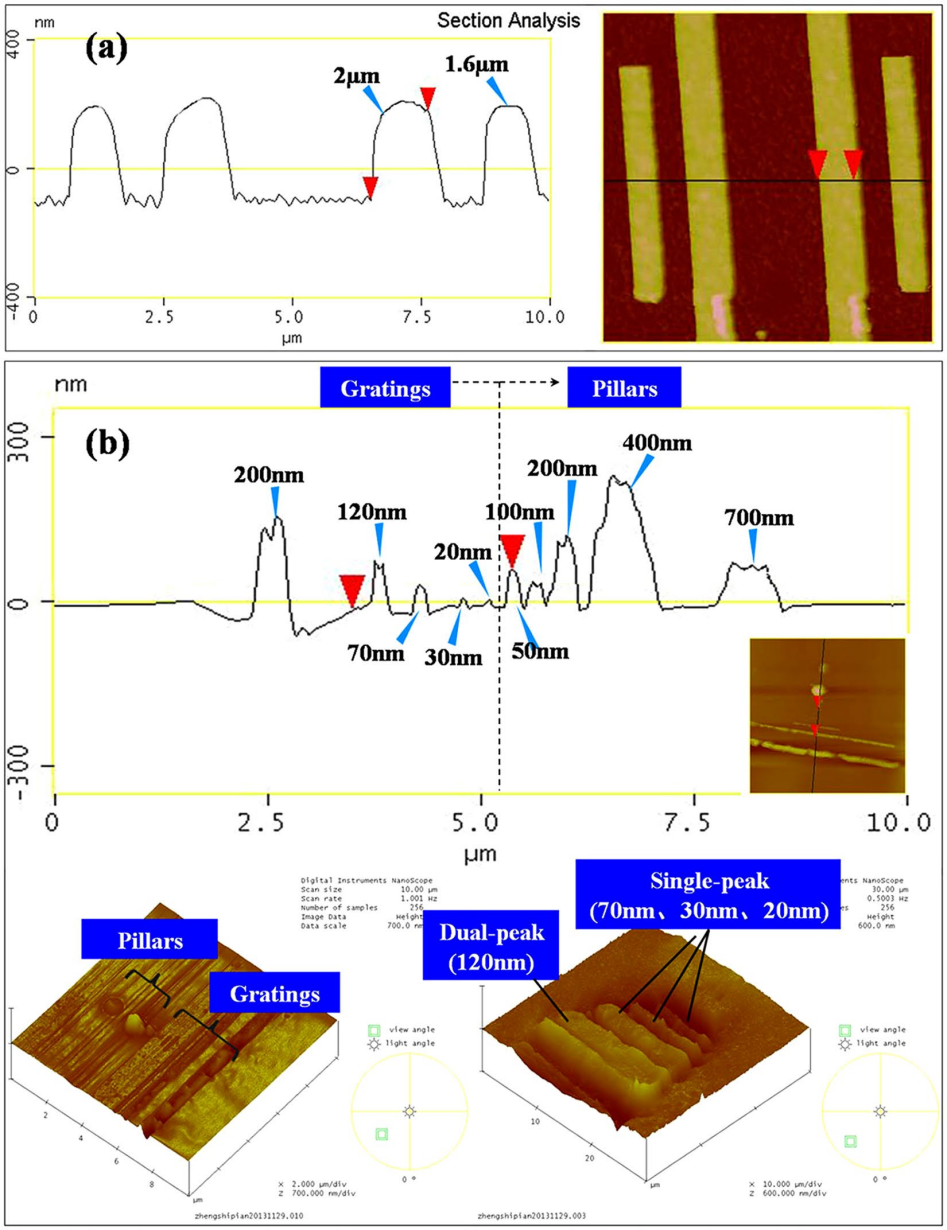
The filling peak of 996 kD PMMA at different scales. (**a**) The single-peak filling mode of PMMA at micron scale. (**b**) The filling peaks at a scale from sub-microns to molecular confinement.

Figure 2a shows the filling peaks of 996 kD PMMA into a 1.6 µm wide pore and a 2 µm wide pore. The peaks were obtained by using AFM. Results have shown that the filling of PMMA into a micro scale cavity (or a pore) has exhibited a single-peak mode. Experiments have also shown that the single-peak filling mode can be observed as the cavity width is above 700 nm. This filling mode means the elastic-plastic character domains the deformation behavior of solid PMMA at this scale. It is well-known for the replication of micro scale structures. We called this as “solid” filling mode.

As the cavity size reduces to 400 nm, the dual-peak filling mode is observed (Fig. 2b). The solid state polymer deforms into the cavity (or the pore) with two peaks form near the cavity sidewalls. The dual-peak filling is also observed as the cavity width further decreases to 200 nm and 120 nm. The dual peak filling is also observed within the pores with a diameter under 700 nm and above 100 nm. In a pore, the “dual-peak filling” means that the peak forms around the pore sidewall. The profile of the polymer deformation is similar with a “volcanic shape” within the pore. Some scholars have reported the dual-peak filling mode of molten polymer^8–16^. The capillary force of molten fluid has been seen as the cause of the dual-peak. For solid state polymer, the “dual-peak” has been observed as the aspect ratio of the cavity is far more than 1.0. But the cause of the each peak of the “dual-peak” is actually similar with the single-peak mentioned above. Elastic-plastic character still domains the deformation. The two peaks is just caused by the extensional stretching which means there are not enough deformation force to support the form of a peak far from the cavity sidewall. However, in a submicron cavity with an aspect ratio of 1.0, the form of dual-peak has rarely been reported. In our former work, the dual-peak filling mode of solid state poly(methyl methacrylate) (PMMA) at a scale from nanometer to submicron has been discussed^14^. This filling mode is taken as the “fluid-like” behavior shown by polymeric solid at this scale. The cause of this deformation can be attributed to the domination of surface force and viscous force in the motion of the polymeric chain^9, 10, 14^.

As the cavity and pore sizes further decrease to below 70 nm, the filling behaviors of PMMA into the cavities or pores become “near-confinement scale deformation” and “confinement scale deformation”. Unlike the reported fluid-like behavior of solid polymer^18, 19^ the PMMA has been found exhibited “solid state” character at this scale. Figure 2b shows the filling peak of solid PMMA as the pore and the cavity sizes are 70 nm, 50 nm, 30 nm and 20 nm, respectively. It can be seen that the filling peaks are all single-peak. The results mean that the elastic-plastic character domains the deformation of PMMA at this scale. The fluid-like behavior which is observed at nanometer scale to submicron scale disappears. This phenomenon is different from the experimental results for PS. It has been found that the resistance to deformation of PS has a markedly reduction at molecular confinement scale. And the deformation of PS during squeeze flow has an obvious acceleration. This phenomenon of PS is attributed to the local entanglement depletion and shear thinning induced by high shear rate.

This solid-fluid-solid filling behavior of PMMA at a scale from microns to molecular confinment also can be exhibited by the moduli, hardnesses and stress-strain curves. The following sections will give these experimental results.

### Modulus and Hardness

**Figure 3:**
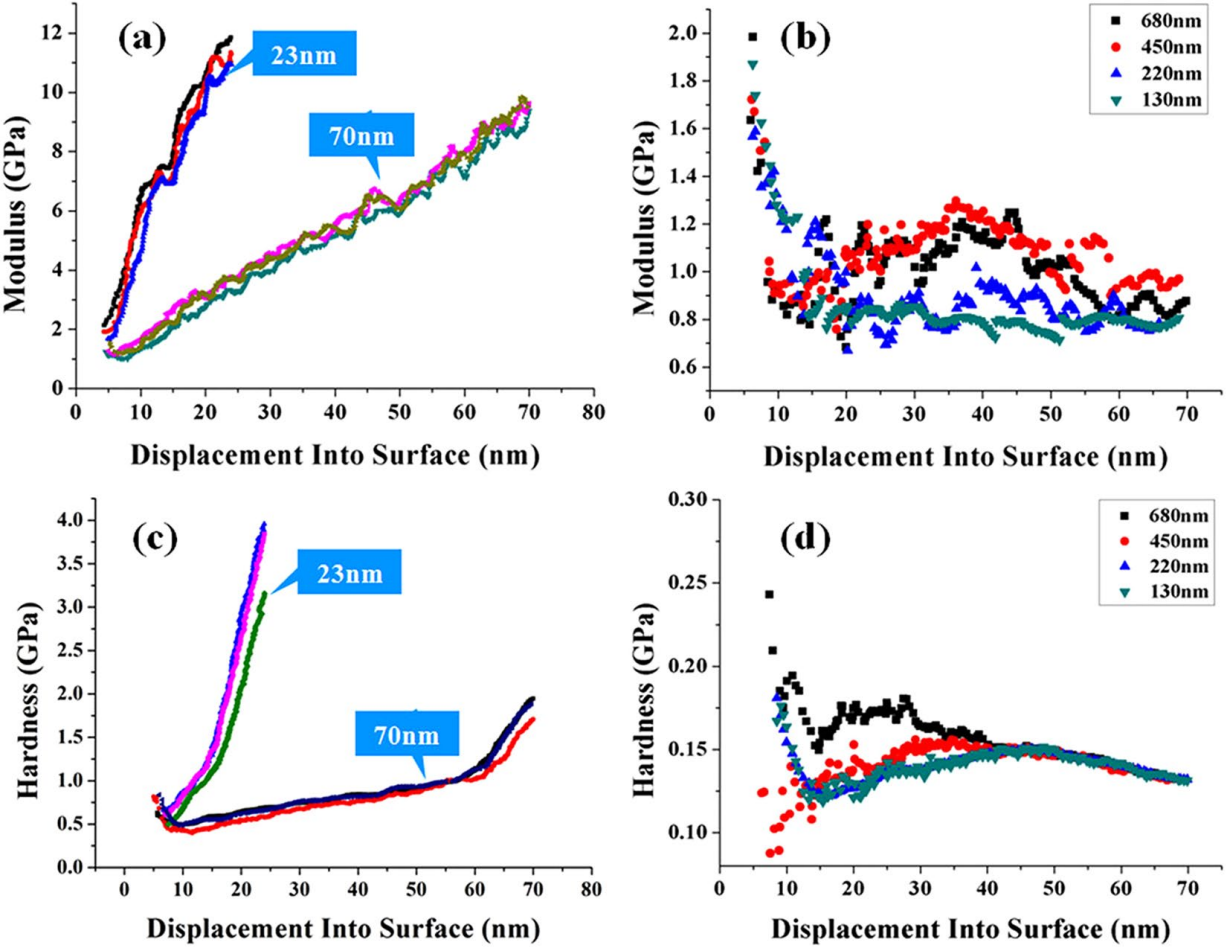
The modulus and hardness curves of 996 kD PMMA at different scales. (**a**) The moduli at 23 nm and 70 nm scales. (**b**) The modulus at 130 nm, 220 nm, 450 nm and 680 nm. (**c**) The hardnesses at 23 nm and 70 nm. (**d**) The hardnesses at 130 nm, 220 nm, 450 nm and 680 nm.

In the 996 kD PMMA used in our experiments, the deformation scale has an obvious effect on the modulus and hardness at 20 nm to 70 nm. We measure the moduli and hardnesses of 23 nm, 70 nm, 130 nm, 220 nm, 450 nm and 680 nm films by using nano-indentation technique at 110 °C. The ratios of the film thickness to the chain length of PMMA are 0.75, 2.3, 4.3, 7.2, 14.8 and 22.3, respectively. Figure 3a shows the surface modulus of 23 nm and 70 nm films. For each thickness, the modulus has been measured three times. It can be seen that, for 23 nm and 70 nm films, their moduli will increase to follow the increase in indentation depths. The increase of 23 nm is more pronounced than that of 70 nm. The top value of the modulus has reached about 12 GPa which is approximate 5–6 folds of the bulk modulus of PMMA. The dramatic rise of modulus means that the resistance to deformation increases. The elastic-plastic mechanical character will become more obvious than normal state at molecular confinement scale and near confinement scale. Figure 3b shows the moduli of 130 nm, 220 nm, 450 nm and 680 nm films. The experiments have been repeated four times. Its changing trend is contrary to that of 23 nm and 70 nm. Their changing trend all shows a pronounced softening behavior. The moduli sharply decrease by an order of 50–70% as the indentation depth increases to 10 nm-20 nm, and the values retain steady as the indentation depth continues to increase.

Figure 3c shows the hardnesses of 23 nm and 70 nm films. The changing trends are similar with that of the modulus. The hardness of 23 nm film increases sharply from 0.5 GPa to about 3 GPa-4 GPa on the order of 600% to 800%. The hardness of 70 nm also exhibits a pronounced increase on the order of approximate 400%. The increase in surface hardness means that the resistance to deformation increases, and the filling of a small gap becomes difficult. The “solid-state” characters will become more obvious. The increase in modulus of 70 nm film has a slight decrease as the indentation depth increases from 0 to 10 nm. And an acceleration of modulus increase has been observed as the indentation depth increases from 60 nm to 70 nm. As compared to the curves of 23 nm, the change has exhibited the difference in hardness between the confinement scale and the near confinement scale. The hardnesses of 130 nm, 220 nm, 450 nm and 680 nm films are shown in Fig. 3d. The softening behaviors are also observed. The surface hardness has decreased by an order of 40–60% as the indentation depth increases to 10 nm-20 nm. Then, the hardness retains steady at this value. The low surface hardness and modulus can obviously reduce the resistance to deformation of molecular chain.

### Stress versus Strain Relationship

**Figure 4:**
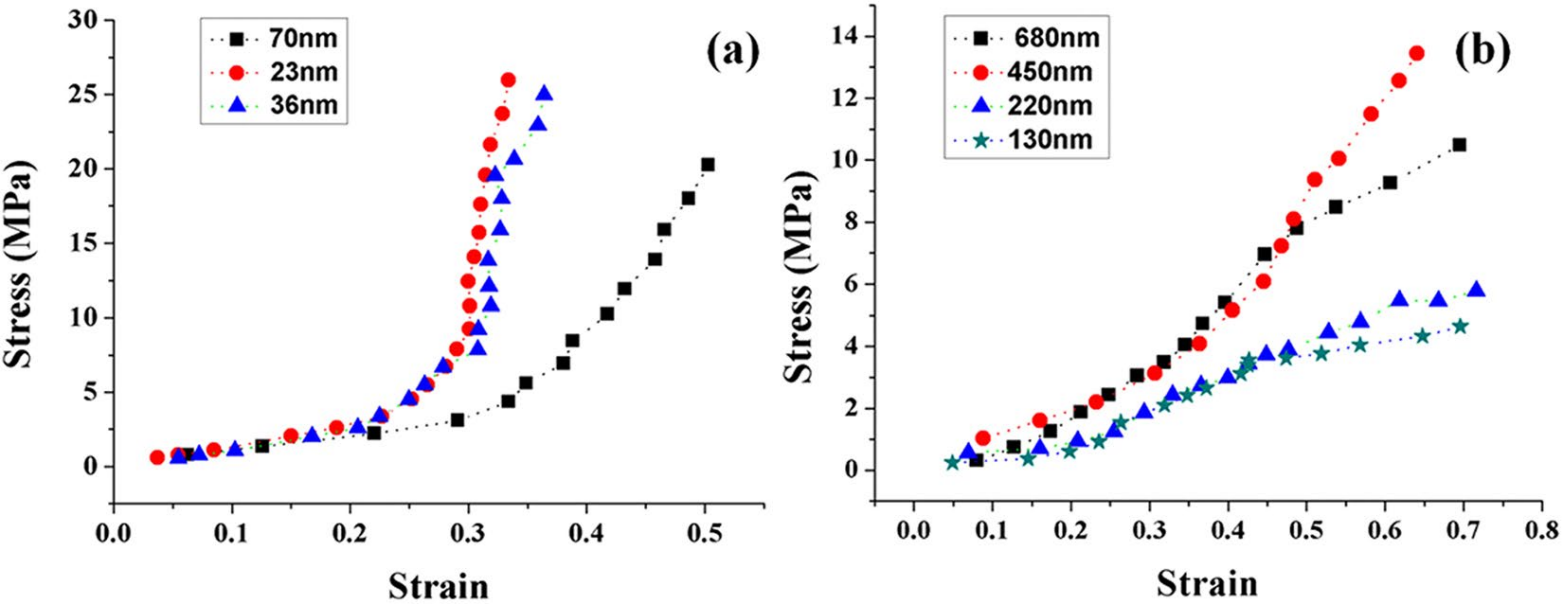
Stress-strain curves at different scales. (**a**) The stress-strain relationships of 996 kD PMMA at 23 nm, 36 nm, and 70 nm scales. (**b**) The stress-strain curves of 996 kD PMMA at 130 nm, 220 nm, 450 nm, and 680 nm scales.

To highlight the anomaly in the mechanical behavior of 996 kD PMMA at near-confinement scale and confinement scale, we measure the stress versus strain for the films of 23 nm, 36 nm, 70 nm, 130 nm, 220 nm, 450 nm, and 680 nm at 110 °C. Figure 4a shows the three values of 70 nm, 36 nm and 23 nm (black, blue and red traces, respectively). The ratios of the film thickness to the chain length of PMMA are 2.3, 1.2 and 0.75. For 72 nm film, as the strain increases from 0 to about 0.3, the stress increases slightly. As the strain increases above 0.3, the stress exhibits a sharp increase. This phenomenon means that the resistance to deformation of 996 kD PMMA has a dramatic increase at 70 nm scale. At 36 nm and 23 nm scale, the stress exhibits an obvious increase as the strain increases from 0.2 to 0.3. As the strain increases from 0.3 to 0.35, the stress has a very sharp increase of nearly 270%, which extend the trend of an increased hardening effect at molecular confinement scale and near confinement scale. Differs from the bulk deformation behavior, the transition from elastic deformation to plastic yield is not clear. The hardening emerges just after the elastic deformation process. We think the hardening effect is the cause of the increase in modulus and hardness shown in Fig. 3a,c. This measured stress-strain response cannot be attributed to the phenomena, such as shear thinning24, entanglement depletion25, and wall slip26. Conventional research think that the high shear rate can induce local entanglement depletion and shear thinning at small scales, which results in the “fluid-like” behavior of polymeric solid27,28. However, the stress-strain curves shown in Fig. 4a are contrary to those studies. The 996 kD PMMA solid has exhibited a hardening state at the molecular confinement scale and near confinement scale under large strain deformation in narrow gaps. We think this phenomenon may be caused by the ordering and bridging effects, the localized stress concentration during the large strain deformation. The relaxation and extension time and space have been confined, which dramatically increase the entanglement of molecular chains and results in localized hardening.

Figure 4b shows the four values of 130 nm, 220 nm, 450 nm and 680 nm (green star, blue triangular, black square and red circular, respectively). For 130 nm and 220 nm film, as the strain increases from 0 to about 0.45, the increase of stress is steady. A softening behavior is observed as the strain increases above 0.4, which shows the reduction of the resistance to deformation. For 450 nm film, the softening behavior is also observed as the strain increases above 0.45. The increase of stress is sharper than that of 130 nm and 220 nm film as the strain increases from 0 to 0.45. For 680 nm film, the softening process still emerges, but not obvious. The curves shown in Fig. 4b exhibits the trend of an increased softening effect as the scale reduces from submicron scale to near nanometer. The softening trend accords with the results of dual-peak filling, the modulus and the hardness change at this scale.

### Molecular Weight

**Figure 5:**
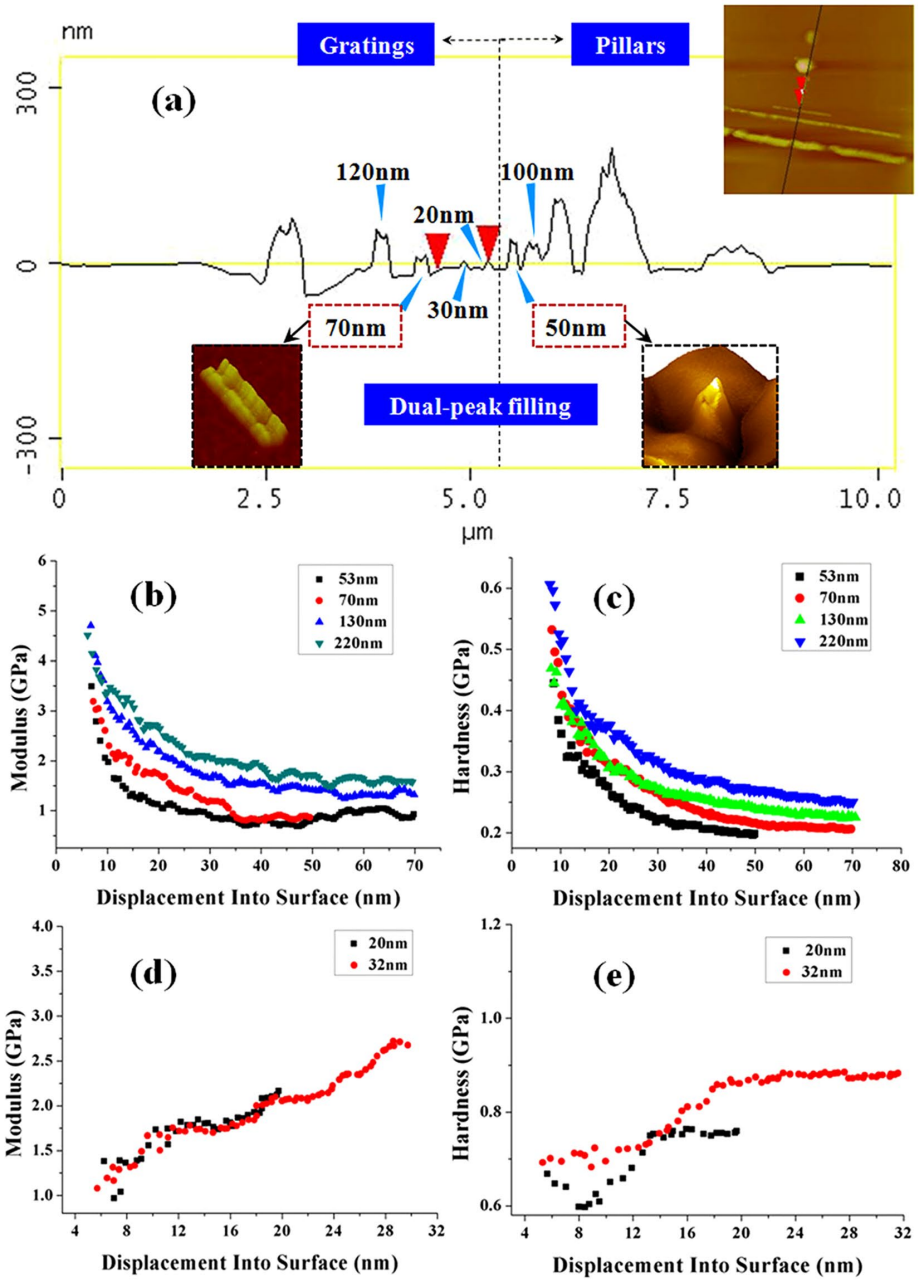
The mechanical behavior of 100 kD PMMA. (**a**) The filling peaks of 100 kD PMMA at the scale from sub-microns to near confinement scale. (**b**) The moduli of 100 kD PMMA at 53 nm, 70 nm, 130 nm, and 220 nm scales. (**c**) The hardnesses of 100 kD PMMA at 53 nm, 70 nm, 130 nm, and 220 nm scales. (**d**) The moduli of 100 kD PMMA at 20 nm and 32 nm scales. (**e**) The hardnesses of 100 kD PMMA at 20 nm and 32 nm scales.

We also study the influence of molecular weight on filling modes and mechanical properties of PMMA. For 100 kD PMMA, the filling peaks are shown in Fig. 5a. Dual-peak filling behavior is still observed as the scale decreases to 50 nm. As the gap size reduces to below 30 nm, single-peak filling is observed. The phenomenon is similar with that of 996 kD. As the scale reduces to near-confinement scale (about 2–3 folds of molecular length), PMMA exhibits elastic-plastic dominant character. As the scale reduces to submicrons but above near-confinement scale, PMMA exhibits visco-dominant character. The modulus and hardness curves shown in Fig. 5b,c also exhibit the trend. A softening behavior is observed as from 53 nm to 220 nm scales. This softening behavior is similar with that exhibited by 996 kD PMMA at a scale above 100 nm. The softening effect can reduce the resistance to deformation of 100 kD PMMA, which may cause the dual-peak filling.

Figure 5d,e show the moduli and hardnesses as the size reduces to 32 nm and 20 nm. The moduli have obvious increases. Their top values have reached about 2–2.7 GPa which is approximate 1–1.5 folds of initial value. The hardness of 20 nm film exhibits an decrease trend as the indentation displacement increases from 0 to nearly 8 nm. As the indentation displacement increases from 8 nm to about 14 nm, the value of hardness exhibits an sharp increase. As the displacement increases further, the increase in hardness becomes slight. The initial decrease has not been observed for 32 nm film. Before the indentation displacement increases to about 19 nm, its hardness increases pronouncedly. As the indentation displacement increases above 19 nm, the increase in hardness also becomes slight. This phenomenon is different with that of 996 kD PMMA. According to our results, 100 kD PMMA has smaller deformation resistance at nanoscale. Hence, decreasing the molecular weight can be helpful in increasing filling accuracy of nanostructures.

## Conclusions

Our results show the filling peaks of PMMA with large molecular weight change from single-peak to dual-peak as the gap size decreases from microns to submicrons. At submicron scale, the modulus, the hardness and the stress-strain curve of PMMA all exhibit a softening behavior which means that the resistance to deformation reduces pronouncedly. The dual-peak filling and the softening behavior indicate the polymeric solid will also exhibits some “viscous-dominant” characters of melt polymer. As the scale reduces to near confinement and molecular confinement, the modulus, the hardness and the stress-strain curve all show a hardening behavior. The resistance to deformation sharply increases. The filling peak becomes single-peak which exhibits the elastic-plastic dominant character. The hardening behavior is contrary to the conventional studies which obtains the results that shear thinning, entangled depletion, and shear band *et al*. will cause the reduction in mechanical stiffness and accelerate the deformation at molecular confinement scale. Experiments also show the molecular weight can affect the filling behavior. As the molecular weight is 100 kD, the dual-peak filling is still observed as the gap size reduces to 50 nm, and we have not observed the hardening behavior at this scale. According to our results, to replicate nanostructures by using the thermoplastics with low molecular weight can be useful in improving replication accuracy due to the visco-dominant behavior of this kind of polymer. The results have implications for the study of solid polymer deformation behavior during micro/nano replication or imprint. It is also useful in the fields, such as ultrathin polymer coatings, polymer-based lubricants, as well as the strength and fatigue of layered polymer.
